# Mechanism of Vascular Toxicity in Rats Subjected to Treatment with a Tyrosine Kinase Inhibitor

**DOI:** 10.3390/toxics8030049

**Published:** 2020-07-20

**Authors:** Claudia Reyes-Goya, Álvaro Santana-Garrido, Estefanía Soto-Astacio, Óscar Aramburu, Sonia Zambrano, Alfonso Mate, Carmen M. Vázquez

**Affiliations:** 1 Departamento de Fisiología, Facultad de Farmacia, Universidad de Sevilla, E-41012 Sevilla, Spain; crgoya@us.es (C.R.-G.); asgarrido@us.es (Á.S.-G.); estefaniasotoastacio@gmail.com (E.S.-A.); vazquez@us.es (C.M.V.); 2 Instituto de Biomedicina de Sevilla (IBIS), Hospital Universitario Virgen del Rocío/Consejo Superior de Investigaciones Científicas/Universidad de Sevilla, E- 41013 Sevilla, Spain; 3 Servicio de Medicina Interna, Hospital Universitario Virgen Macarena, E-41009 Sevilla, Spain; oscarab2000@gmail.com; 4 Integrated Cardiometabolic Center, Department of Laboratory Medicine, Karolinska Institutet at Karolinska University Hospital Huddinge, 17177 Stockholm, Sweden; sonia.zambrano.sevilla@ki.se

**Keywords:** arterial hypertension, endothelial dysfunction, tyrosine kinase inhibitor, vascular remodeling

## Abstract

Sunitinib (Su) is a tyrosine kinase inhibitor with antiangiogenic and antineoplastic effects that is recommended therapy for renal cell carcinoma, gastrointestinal stromal tumors, and pancreatic neuroendocrine tumors. Arterial hypertension is one of the adverse effects observed in the treatment with Su. The aim of this work was to deepen our understanding of the underlying mechanisms involved in the development of this side effect. Studies on endothelial function, vascular remodeling and nicotinamide adenine dinucleotide phosphate oxidase (NADPH oxidase) system were carried out in thoracic aortas from rats treated with Su for three weeks. Animals subjected to Su treatment presented with increased blood pressure and reduced endothelium-dependent vasodilation, the latter being reverted by NADPH oxidase blockade. Furthermore, vascular remodeling and stronger Masson trichrome staining, together with enhanced immunofluorescence signal for collagen 1 alpha 1 (Col1α1), were observed in aortas from treated animals. These results were accompanied by a significant elevation in superoxide anion production and the activity/protein/gene expression of NADPH oxidase isoforms (NOX1, NOX2, and NOX4), which was also prevented by NOX inhibition. Furthermore, a decrease in nitric oxide (NO) levels and endothelial nitric oxide synthase (eNOS) activation was observed in aortas from Su-treated animals. All these results indicate that endothelial dysfunction secondary to changes in vascular remodeling and oxidative stress might be responsible for the typical arterial hypertension that develops following treatment with Su.

## 1. Introduction

Antiangiogenic treatment with tyrosine kinase inhibitors (TKIs) has been widely used in cancer therapy in the last years [1]. TKIs effect consist of competing with ATP for the ATP-binding site on protein tyrosine kinases, thus decreasing phosphorylation and cancer cell proliferation. TKIs inhibit epidermal growth factor receptor (EGFR), platelet-derived growth factor receptor (PDGFR), and vascular endothelial growth factor receptor (VEGFR). The variety of commercially available TKIs has improved the therapeutic management of cancer in the last decades. However, these drugs are often associated with adverse side effects, including cardiovascular toxicity and arterial hypertension [2].

Sunitinib (Su) is an orally active multitarget TKI accepted by the U.S. Food and Drug Administration (FDA) and by the European Commission [3]. Su is the first-line choice for metastatic renal cell carcinoma treatment and the second-line one for gastrointestinal stromal tumors (GISTs) and pancreatic neuroendocrine tumors [4,5]. This compound binds to several tyrosine kinase receptors including VEGFR, PDGFR, and the stem cell factor receptor (c-KIT). Similar to other VEGFR inhibitors, arterial hypertension is a common feature associated with Su treatment [6]. Although several mechanisms have been suggested to explain this side effect, the underlying pathophysiological mechanism remains unknown. Reduced nitric oxide (NO) generation, endothelial dysfunction, and capillary rarefaction have been proposed as factors involved in anti-VEGF-induced arterial hypertension [7,8]. Su-induced arterial hypertension has also been related to an increase in endothelin-1 (ET-1) levels [9], and the administration with the ET-1 receptor antagonist macitentan showed attenuation in the elevation of blood pressure [10]. However, these results were not reproduced with bosentan, another blocker of the ET-1 receptor [11]. Additionally, stiffness of large arteries, changes in arterial mechanical properties and fibrosis have been implicated in blood pressure elevation secondary to treatment with TKIs [12,13], suggesting that local vasoconstriction in specific areas might be responsible for the onset of arterial hypertension [11].

Other investigators, in addition to our previous work, have demonstrated that enhanced oxidative stress is one of the relevant alterations in hearts from Su-treated rats, suggesting the important role of reactive oxygen species (ROS) in the vascular dysfunction and hypertension processes surrounding TKI-induced cardiotoxicity [14,15,16]. Our present work furthers our understanding of the possible mechanisms involved in arterial hypertension secondary to treatment with Su. To this purpose, histomorphometric studies were carried out in aortas from Su-treated rats, to check this tissue for vascular remodeling and analyzing the location of type I collagen as a final product of fibrosis. In addition, superoxide anion (O_2_^−^) production, location, activity, and expression of ROS-generating nicotinamide adenine dinucleotide phosphate oxidase (NADPH oxidase), NO levels, endothelial nitric oxide synthase (eNOS) protein/mRNA expression, together with vascular reactivity experiments, were performed in aortas from Su-treated animals and compared with non-treated normotensive Wistar rats.

## 2. Materials and Methods

### 2.1. Animals and Experimental Design

Male Wistar rats (8–10 weeks old) were supplied by the Center for Animal Production and Experimentation (University of Seville, Spain) and kept under standard conditions (23 ± 1 °C, 12h/12h light/dark cycles). All procedures complied with the European Union (EU Directive 2010/63/EU) and the National (RD 53/2013) guidelines and were approved by the competent Institutional Animal Care and Use Committee (approval reference #08/03/2017/034; date of approval: 08 March 2017, issued by Junta de Andalucía, Dirección General de la Producción Agrícola y Ganadera). Two groups of 15 rats each were randomly assigned to: (i) control group with free access to food and tap water; and (ii) rats subjected to treatment with 25 mg Su/kg body weight/day for three weeks. An appropriate solution of Su (Pfizer Inc, 2019) was prepared in tap water and homogenized mixed homogeneously within crushed pellet to form Su-containing pellets. To ensure the correct dosage, the concentration of Su was weekly adjusted according to each animal’s body weight and average daily food intake.

### 2.2. Blood Pressure and Tissue Preparations

During the experimental period, blood pressure, heart rate, and body weight were continuously monitored, as previously described [14]. Upon treatment completion, animals were injected with 75 mg/kg ketamine + 10 mg/kg diazepam (i.p.), the intact thoracic aorta was removed and ice-cold 0.9% saline solution was used to wash the tissue. The connective tissue around the aorta was removed and the vessel was then cut into pieces. The superior portion (including the aortic arch) was immediately frozen in liquid nitrogen and stored at −80 °C until use for detection of mRNA/protein expression. The rest was employed for functional studies (vascular reactivity) and dihydroethidium (DHE) assay and histomorphometric studies. All animals were routinely sacrificed between 9:00 to 10:00 h to minimize diurnal variation.

Aorta tissue was homogenized in radioimmunoprecipitation assay (RIPA) buffer with a micro-pestle motor-driven tissue homogenizer (Heidolph Instruments, Schwabach, Germany) for Western blot studies. After centrifugation at 2000× *g* for 10 min at 4 °C, the supernatant was stored at −80 °C and the pellet was discarded. The Bradford method was used for determining protein concentration [17] (Bio-Rad Protein Assay. Bio-Rad Laboratories, USA).

### 2.3. Measurement of the Levels of Superoxide Anion and Nitric Oxide Concentration

The superoxide-sensitive fluorescent dye dihydroethidium (DHE; MedChemExpress, HY-D0079) was used to estimate the level of O_2_^−^ as previously reported [18]. Thoracic aortas were embedded in Tissue-Tek® O.C.T.™ (Sakura Finetek, 4583) and frozen sections were cut into 10 µm thick sections with a cryostat (Leica CM1510 S, Leica Biosystems). Sections were thawed, incubated with DHE (10 mM) at 37 °C for 10 min and with 4’,6-diamidino-2-phenylindole (DAPI) Fluoromount-G^®^ (SouthernBiotech, 0100-20). Serial sections were treated with either 10 µmol/L VAS2870 (pan-inhibitor of NOX isoforms; Merk Millipore, 492000), 0.1 µmol/L GKT136901 (NOX1 and NOX4 inhibitor, Merk Millipore, 5340320001), 0.5 µmol/L ML171 (specific NOX1 inhibitor; Sigma Aldrich, 175226); or 100 µmol/L L-NAME (eNOS inhibitor; Sigma-Aldrich, N5751) for 30 min prior to incubation with DHE. Preincubation with 100 U/mL polyethylene glycol-conjugated SOD (PEG-SOD; Sigma Aldrich, S9549) for 10 min at 37 °C was carried out to confirm the specificity of staining. All sections were examined on a fluorescence microscope (Olympus BX61. Olympus Corporation) and photographed with a color digital camera (Olympus DP73. Olympus Corporation). O_2_^−^ production was estimated from the ratio ethidium/DAPI fluorescence signal using ImageJ (Version 2.0.0-rc-69/1.52p. National Institutes of Health, Bethesda, MD). Nitrite and nitrate levels in aorta homogenates were estimated by the Griess method [19] in the absence and presence of NOX inhibitors in the same final concentration as described above.

### 2.4. Measurement of NADPH Oxidase Activity

Aorta homogenates were used to determine NADPH oxidase activity by lucigenin-enhanced chemiluminescence, as previously reported [20]. In order to confirm the isoform involved in the production of superoxide anions (O_2_^−^), homogenate samples were preincubated for 5 min at 37 °C with VAS2870, GKT136901, ML171, and L-NAME at the same final concentrations stated in Section 2.3.

### 2.5. RNA Extraction and Real-Time PCR

Frozen aortas were used to extract total RNA and retro-transcribed as previously described [21]. The generated cDNAs were used for real-time PCR with SYBR Green^TM^ (Roche, 04673514001) reactions in a LightCycler1 480 Real-Time PCR System (Roche Diagnostics, Madrid, Spain). Primer sequences (Biomedal, Seville, Spain) used in this study are detailed in Table 1, including glyceraldehyde 3-phosphate dehydrogenase (GAPDH) that was used as an endogenous control reference. Data were analyzed and quantified with the 2^−ΔΔCt^ method [22]. 

### 2.6. Western Blotting

Following SDS-PAGE, proteins were transferred to nitrocellulose membrane and incubated with specific antibodies: mouse monoclonal anti-TGF-β1 (3C11) (Santa Cruz Biotechnology, Santa Cruz, CA, USA; 1:3000 dilution); rabbit polyclonal anti-MOX1 (H-75) (Santa Cruz Biotechnology, Santa Cruz, CA, USA; 1:1000 dilution); rabbit monoclonal anti-NOX2 (Epitomics-Abcam, Burlingame, CA, USA; 1:8000 dilution); rabbit monoclonal anti-NOX4 (Epitomics-Abcam, Burlingame, CA, USA; 1:7500 dilution); mouse monoclonal anti-NOS3 (A-9) (Santa Cruz Biotechnology, Santa Cruz, CA, USA; 1:2000 dilution); purified mouse anti-eNOS (pS1177) (BD Transduction Laboratories, 1:2000 dilution); mouse monoclonal anti-p-NOS3 (pt495.33) (Santa Cruz Biotechnology, Santa Cruz, CA, USA; 1:2000 dilution); mouse monoclonal anti-3-nitrotyrosine (Santa Cruz Biotechnology, Santa Cruz, CA, USA; 1:1000 dilution). Mouse monoclonal anti-β-actin (Santa Cruz Biotechnology, Santa Cruz, CA, USA; 1:20,000 dilution) was also used for protein loading control. Adequate anti-rabbit or anti-mouse secondary antibodies were used following the manufacturer’s recommendations, and signals were revealed with ECL™ Prime Western Blotting System (Amersham, RPN2232) and measured with an Amersham Imager 600 (GE Healthcare Life Science).

### 2.7. Vascular Reactivity

Vascular function was carried out as previously reported [23] using a Panlab organ bath (Harvard Apparatus) and a PowerLab^®^ 8/30 data acquisition system (ADInstruments) coupled to appropriate transducers and controlled by the LabChartTM software (ADInstruments). Endothelium-intact aortic rings were contracted with 10^−9^–3×10^−5^ mol/L phenylephrine (Phe) and relaxed with 10^−9^–3×10^−5^ mol/L acetylcholine (ACh) or 10^−10^–3×10^−6^ mol/L sodium nitroprusside (SNP). Selective NOX1 inhibitor ML171 (2-acetylphenothiazine) and eNOS inhibitor (L-NAME) were preincubated at 10^−4^ mol/L. 

### 2.8. Histomorphometric Studies

Slices from thoracic aortas were stained with hematoxylin and eosin and photographed with an Olympus BX41 microscope. The area and thickness of tunica media and the lumen area were calculated using Image J (Version 2.0.0-rc-69/1.52p. National Institutes of Health, Bethesda, MD). Additional slices were stained with Masson’s trichrome to determine qualitative total collagen content following the instructions from the Sigma-Aldrich kit HT15-1KT. Images were captured and processed with bright field of the microscope Zeiss Axio Observer Z1 using bright field and the 10× objective.

### 2.9. Immunofluorescence Studies

Double immunofluorescence was carried out on frozen sections using the following antibodies: α-smooth muscle actin-Cy3 (α-SMA) (Sigma Aldrich, 1:1000 dilution); sheep polyclonal anti-Collagen 1α1 (Alexa Fluor; 1:200 dilution); rabbit polyclonal anti-CD31 (Epitomics-Abcam, Burlingame, CA, USA; 1:100 dilution); rabbit polyclonal anti-MOX1(H-75) (Santa Cruz Biotechnology, Santa Cruz, CA, USA; 1:200 dilution); goat polyclonal anti-CD31/PECAM-1 (Alexa Fluor; 1:500 dilution). After incubation, sections were treated with appropriate anti-rabbit, anti-goat or anti-sheep fluorescent secondary antibodies. Nuclei were also stained with DAPI during secondary antibody incubation. Random aortic rings were captured and processed with a Zeiss Axio Observer Z1 Inverted Phase Contrast Fluorescence Microscope using the 20×, 40× or oil 100× objective.

### 2.10. Statistics

Results were evaluated using GraphPad Prism version 5.01 (GraphPad Software Inc., San Diego, CA, USA, 2007) and expressed as the mean ± S.E.M. Differences in mean values between groups were analyzed with the unpaired, two-tailed Student’s *t*-test and considered statistically different at *p* < 0.05. Data from ACh-mediated vasodilation, including preincubation with ML171, DHE, NADPH oxidase, and NO assays were analyzed by one-way ANOVA followed by the Tukey’s multiple comparisons test and considered statistically different at *p* < 0.05.

## 3. Results

### 3.1. General Characteristics of Animals

**Figure 1:**
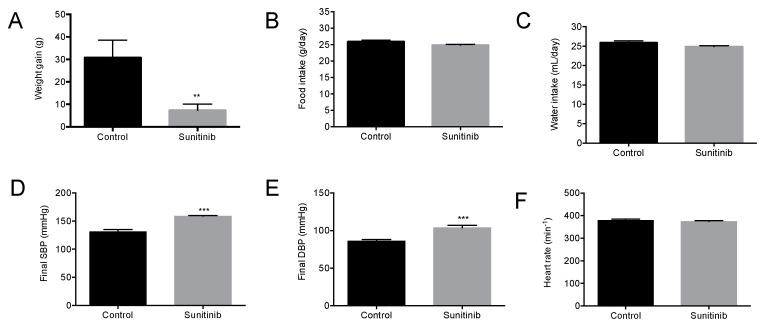
(**A**) Weight gain, (**B**) food intake, (**C**) water intake, (**D**) systolic blood pressure, (**E**) diastolic blood pressure, and (**F**) heart rate in control and sunitinib (Su)-treated (25 mg/kg/day) animals. Values are expressed as mean ± S.E.M. of 15 animals per group. ** *p* < 0.01, *** *p* < 0.001 vs. control group.

Upon three-week experimental period completion, a significant decrease in weight gain was observed in treated animals when compared with control group (Figure 1A), despite no changes in food/water intake between both groups of study (Figure 1B,C). In addition, Su-treated animals presented with enhanced final blood pressure values (Figure 1D,E). On the other hand, heart rate values were similar in both groups (Figure 1F).

### 3.2. Endothelial Dysfunction in Su-treated Rats

**Figure 2:**
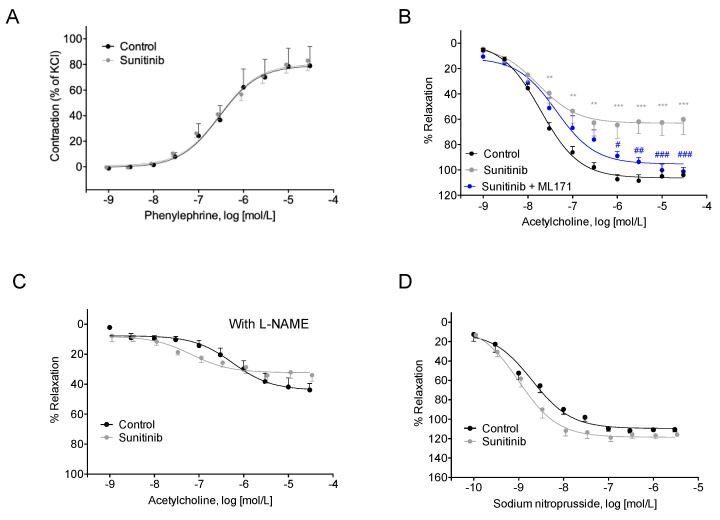
Dose-response curve of (**A**) vasoconstriction mediated by phenylephrine (Phe) (10^−9^–3 × 10^−5^ mol/L); (**B**) ACh-mediated (10^−9^–3 × 10^−5^ mol/L) vasodilation, including preincubation with ML171 (10^−4^ mol/L); (**C**) ACh-mediated vasodilation in the presence of L-NAME (Nω- nitro-L-arginine methyl ester (10^−4^ mol/L); and (**D**) vasorelaxation response to sodium nitroprusside (SNP) (10^−10^–3 × 10^−6^ mol/L) in phenylephrine (Phe)-precontracted vessels from control and sunitinib-treated (25 mg/kg/day) animals. The results correspond to mean ± S.E.M. of at least six experiments (*n* = 6–12). Results are expressed as relative percentages of the maximum contraction induced by 60 mmol/L KCl (**A**), or to the contraction induced by a submaximal dose of Phe (**B**–**D**). ** *p* < 0.01; *** *p* < 0.001 vs. control group, # *p* < 0.05, ## *p* < 0.01, ### *p* < 0.001 vs. Su group.

Vascular reactivity studies showed no changes between both groups of rats regarding the vasoconstrictor response to phenylephrine (Phe) (Figure 2A). On the other hand, endothelium-dependent vasodilation in response to acetylcholine (ACh) was significantly lower in aortic rings from treated animals (Figure 2B); thus, a significant decrease in ACh-induced maximal vasorelaxation response (E_max_) was observed in this group when compared with the control group (63 ± 3.8% vs. 106 ± 1.7%, respectively; *p* < 0.01). In contrast, EC_50_ values for ACh-induced vasodilation were similar in both experimental groups (7.72 ± 0.06 vs. 7.76 ± 0.24 for control and Su, respectively). Interestingly, when aortic rings from Su-treated rats were preincubated with ML171 (an inhibitor of NOX1 isoform of NADPH oxidase), the E_max_ for endothelium-dependent relaxation reached values similar to those of the control group (95.3 ± 2.98%), while EC_50_ was unaffected by the inhibitor (7.36 ± 0.11). When studies on ACh-induced vasodilation were done in the presence of 10^−4^ mol/L L-NAME (Nω- nitro-L-arginine methyl ester, an inhibitor of NO synthesis), E_max_ values dropped to similar levels in both cases (44 ± 2.88% and 32.3 ± 1.93% for control and treated rats, respectively; Figure 2C). Results concerning endothelium-independent vasorelaxation did not show changes between control and Su groups in either E_max_ (109 ± 2.4% vs. 118.5 ± 3.1%) or EC_50_ (8.7 ± 0.09 vs. 8.9 ± 0.09) parameters in response to nitric oxide donor sodium nitroprusside (SNP; Figure 2D).

### 3.3. Vascular Remodeling and Fibrosis

**Figure 3:**
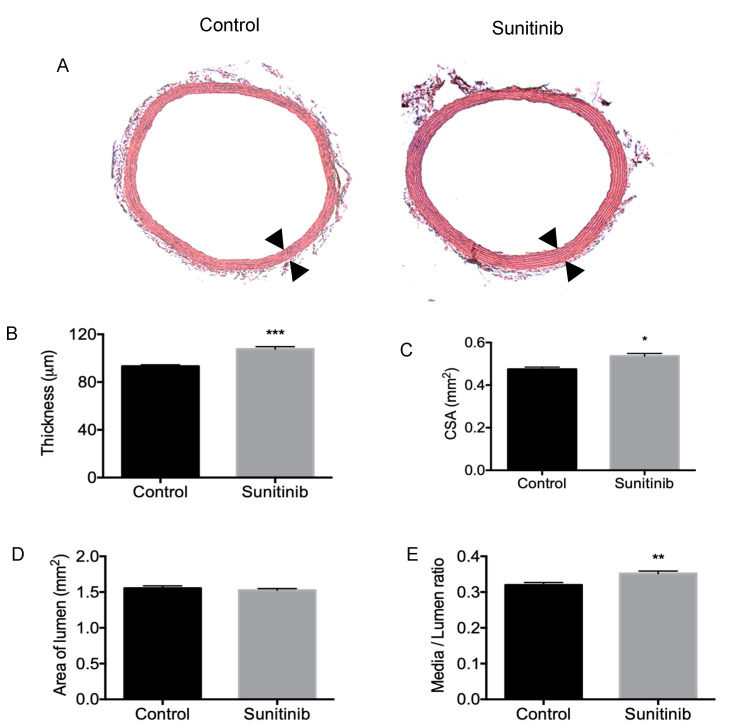
Aorta thickness (**A** and **B**; delimited by arrowheads), cross-sectional area (CSA) of tunica media (**C**), lumen area (**D**) and media/lumen ratio (**E**) in control and Su-treated (25 mg/kg/day) animals. Magnification: 10×. Values are expressed as mean ± S.E.M. of six animals per group. * *p* < 0.05; ** *p* < 0.01; *** *p* < 0.001 vs. control group.

Morphometric studies revealed that the thickness of tunica media (Figure 3A,B) and the cross-sectional area (CSA; Figure 3C) increased in Su-treated rats when compared with control rats. Since the luminal area showed no changes between groups (Figure 3D), a rise in the media/lumen ratio was found in Su-treated rats (Figure 3E), thus indicating the presence of vascular remodeling in these hypertensive animals.

**Figure 4:**
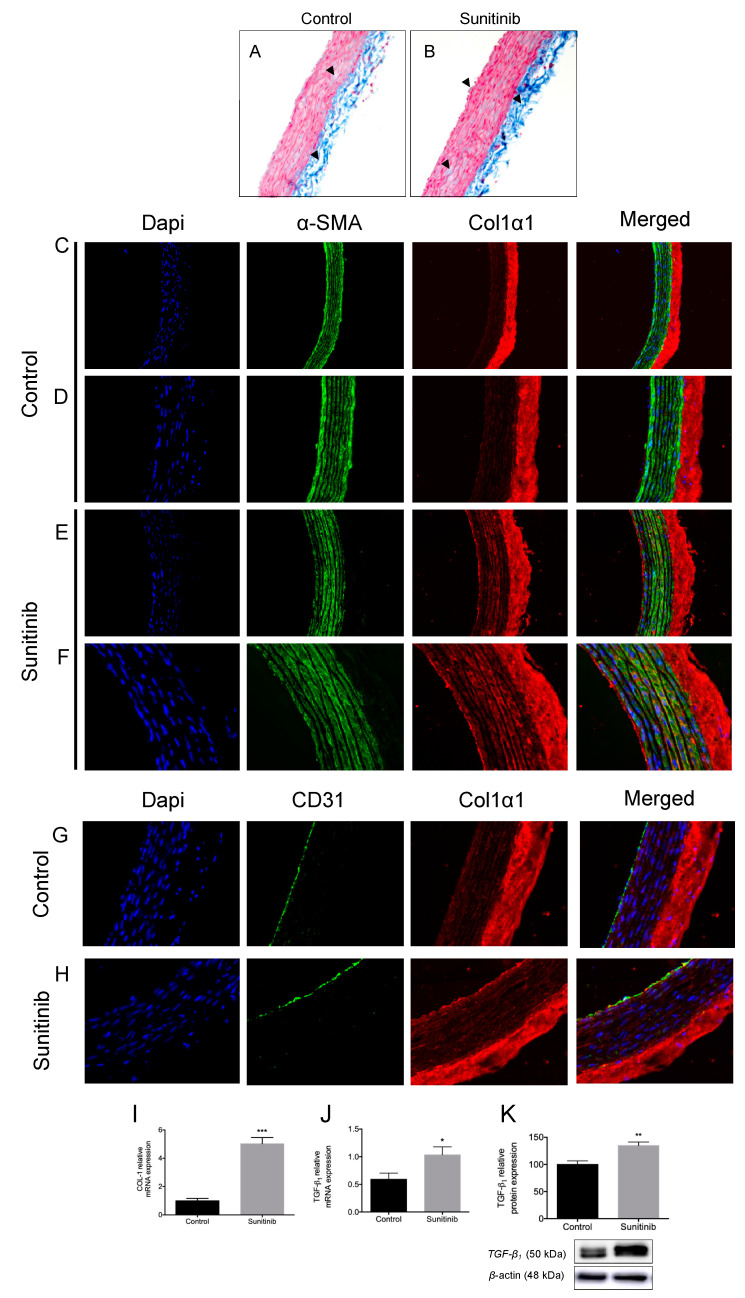
Masson’s trichrome staining for collagen fibers in blue (**A**,**B**, marked with arrowheads), double immunostaining of collagen 1 alpha 1 (Col1α1) (red) and α-smooth muscle actin (α-SMA) from vascular smooth muscle cells (VSMCs) (green) (**C**–**F**), and Col1𝛼1 (red) and CD31 (green) (**G**,**H**), and protein/mRNA expression of Col1 (**I**) and TGF-β1 (**J**,**K**), in aortas from control and sunitinib-treated (25 mg/kg/day) animals. Magnification 10× for Masson’s trichrome staining. Magnifications: 10× (**A**,**B**), 20× (**C**,**E**) and 40× (**D**,**F**–**H**). Values are expressed as mean ± S.E.M. of at least 6 animals per group (*n* = 6–8). * *p* < 0.05; ** *p* < 0.01; *** *p* < 0.001 vs. control group.

The Masson’s trichrome staining showed a slightly intensified blue coloration in aortas from treated rats, revealing an increased total content of collagen (Figure 4A,B). Collagen 1 alpha 1 (Col1α1) isoform is per se expressed in the outer layer of the aortic vascular tissue in both groups, mostly in the adventitia (Figure 4C,D). Nevertheless, aortic rings from Su-treated rats also showed an intense immunofluorescence staining with collagen deposition in the media and intima layer (basement membrane and the extracellular matrix of endothelial cells) (Figure 4E–H). In addition, the mRNA expression of Col1 and the protein and gene expression of TGF-β were also increased in aortas from treated rats (Figure 4I–K), thus suggesting a correlation between Col1α1 immunofluorescence and the expressions of these fibrosis-related components.

### 3.4. Assessment of Vascular Oxidative Stress

**Figure 5:**
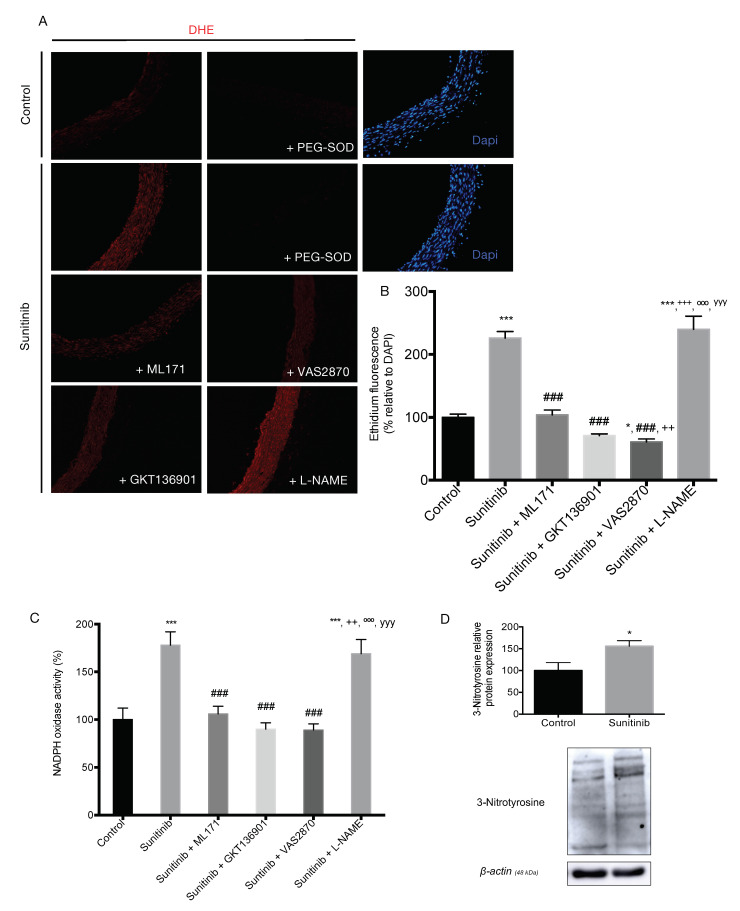
Superoxide anion production in thoracic aortas. (**A**) Representative dihydroethidium (DHE) staining, (**B**) superoxide anion quantification, (**C**) NADPH oxidase activity in aorta homogenates, and (**D**) 3-nitrotyrosine protein expression, in aortas from control and sunitinib-treated (25 mg/kg/day) animals. Magnification: 10×. Values are expressed as mean ± S.E.M. of at least four animals per group (*n* = 4–8). * *p* < 0.05, *** *p* < 0.001 vs. control group; ## *p* < 0.01, ### *p* < 0.001 vs. Su group; ++ *p* < 0.01, +++ *p* < 0.001 vs. Su + ML171 group; ººº *p* < 0.001 vs. Su + GKT136901 group; yyy *p* < 0.001 vs. Su + VAS2870 group.

Dihydroethidium (DHE) imaging showed an increase in superoxide anion (O_2_^−^) production in the aortas of rats subjected to treatment with Su compared to the control group (Figure 5). When aortic ring preparations were preincubated with polyethylene glycol-superoxide dismutase (PEG-SOD, DHE staining disappeared and confirmed the presence of O_2_^−^. Preincubating aortic segments from Su-treated animals with NADPH oxidase inhibitors, namely ML171, GKT136901, and VAS2870, attenuated the superoxide anion formation back to levels measured in the control group. On the other hand, no change in O_2_^−^ production was observed following incubation of rings from Su-treated animals with the eNOS inhibitor, L-NAME (Figure 5A,B).

The activity of NADPH oxidase was increased in aortas from Su-treated rats compared to the control group (Figure 5C). Again, NOX inhibitors reduced the activity of the enzyme in aortas from Su-treated rats in a similar manner as observed with DHE staining. In contrast, L-NAME had no effect on NADPH oxidase activity in Su-treated rats. These results were accompanied by a significant increase in the protein expression of the oxidative stress biomarker, 3-nitrotyrosine, in aortas from Su-treated rats compared to the control group (Figure 5D).

**Figure 6:**
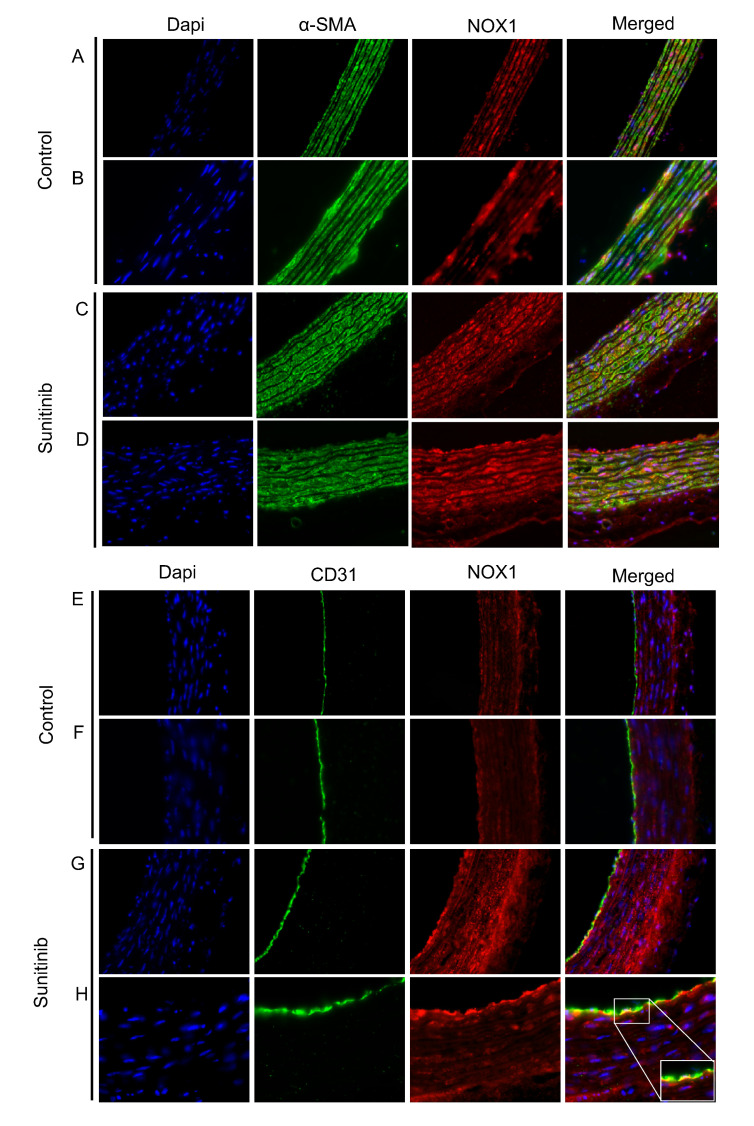

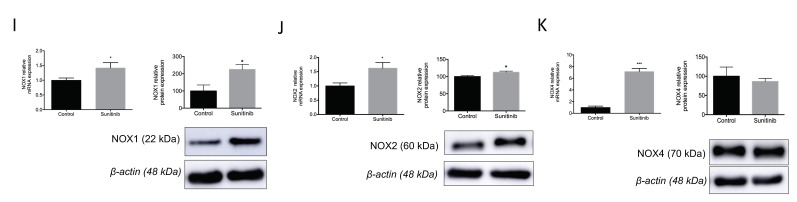
Double immunostaining of NOX1 (red) and α-SMA (VSMCs) (green) (**A**–**D**), and NOX1 (red) and CD31 (green) (**E**–**H**), in aortas from control and sunitinib-treated (25 mg/kg/day) animals. Magnifications: 40× (**A**,**C**,**E**,**G**) and 100× (**B**,**D**,**F**,**H**). (**I**–**K**) Protein and mRNA expression of NOX1 (**I**), NOX2 (**J**), and NOX4 (**K**). Values are expressed as mean ± S.E.M. of eight animals per group. * *p* < 0.05; *** *p* < 0.001 vs. control group.

The double immunofluorescence staining of NOX1 isoform of NADPH oxidase (red) and α-smooth muscle actin (α-SMA) (green) showed that NOX1 is located in VSMCs and the adventitia from control aortas and Su-treated animals, with a stronger co-localization in the latter (Figure 6A–D). The double immunostaining of NOX1 (red) and CD31 (green) showed the co-localization of NOX1 within endothelial cells in aortas from Su-treated rats (Figure 6E–H). Therefore, an increase in the presence of NOX1 was observed in all three vascular layers in aortas from rats subjected to treatment with Su. These results correlated with a rise in the gene and protein expression of NOX1 and NOX2 in aortas from Su-treated (Figure 6I,J). As for the isoform NOX4, although hypertensive animals also showed an upregulation in the gene expression of this NADPH isoform, no changes were observed between both groups in the relative amount of protein estimated from Western blot analysis (Figure 6K).

**Figure 7:**
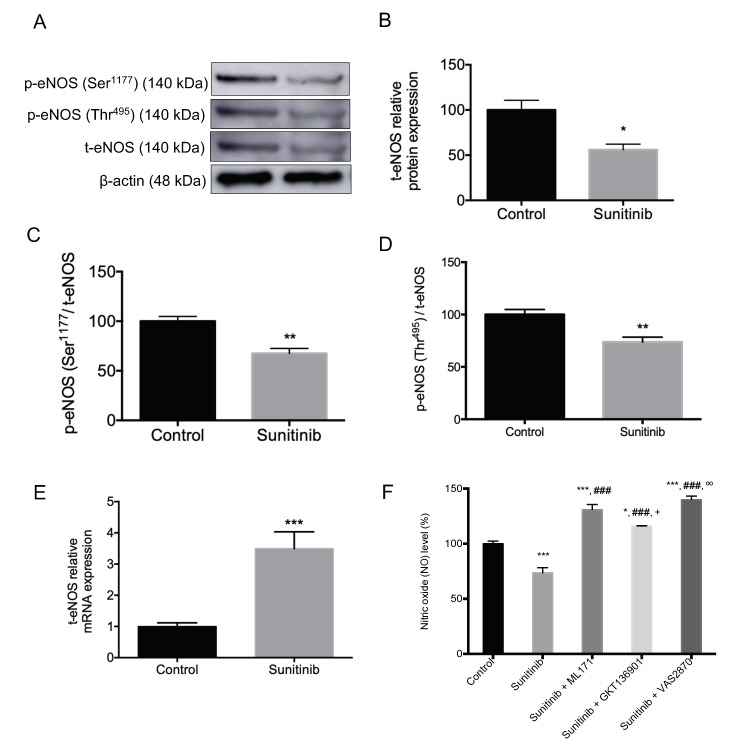
Protein expression of (**A**) p-endothelial nitric oxide synthase (eNOS) (Ser^1177^) and p-eNOS (Thr^495^); (**A**,**B**) total eNOS (t-eNOS); (**C**) ratio p-eNOS (Ser^1177^)/total eNOS; (**D**) ratio p-eNOS (Thr^495^)/total eNOS; (**E**) t-eNOS mRNA expression; and (**F**) NO levels in absence or presence of NOX inhibitors, in aortas from control and sunitinib-treated (25 mg/kg/day) animals. Values are expressed as mean ± S.E.M. of eight animals per group. * *p* < 0.05; ** *p* < 0.01; *** *p* < 0.001 vs. control group.; ### *p* < 0.001 vs. Su group; + *p* < 0.05 vs. Su + ML171 group; ºº *p* < 0.01 vs. Su + GKT136901 group.

Additional experiments revealed a decline in total eNOS protein expression (Figure 7A,B) and phosphorylation of eNOS at Ser^1177^ and Thr^495^ (Figure 7A) in Su-treated group. When the ratio p-Ser^1177^ eNOS/total eNOS and p-Thr^495^ eNOS/total eNOS was determined, a reduction in both parameters was measured in rats subjected to treatment with Su (Figure 7C,D). Furthermore, the gene expression of eNOS was increased in this group of hypertensive animals (Figure 7E). NO levels showed a decrease in aorta homogenates from Su-treated animals, which was reverted to control levels by preincubation with NOX inhibitors (Figure 7F).

## 4. Discussion

Multiple studies have shown a clear correlation between TKIs therapy and the development of cardiovascular toxicity, including arterial hypertension as a common side effect of VEGF inhibitors used in cancer medicine [2]. We and others have focused on enhanced oxidative stress as an important component of cardiotoxicity induced by either Su [14,16], or other TKIs such as VEGFR inhibitor vatalanib and EGFR inhibitor gefitinib [15]. However, since the factors underlying Su-induced arterial hypertension remain unknown [24], here we aimed to gain further knowledge on possible mechanisms triggering Su-induced vascular damage.

Su is administered orally and is used as a first-line treatment in adults with metastatic renal cell carcinoma (mRCC) [5]. The current study was designed in male rats due to the higher frequency of mRCC in men compared with women [25]. In addition, the terminal half-life after oral administration of Su was similar in female and male rats [26]. Our results are in agreement with several studies reporting the use of various antitumor agents [27,28]. Thus, weight gain was negatively affected by Su treatment, although food and water intake remained the same in both experimental groups. Increased levels in systolic and diastolic blood pressures were observed in Su-treated animals, a common feature also observed with other inhibitors of angiogenesis, such as sorafenib (VEGFR inhibitor) and bevacizumab (monoclonal antibody against VEGF) [29,30,31]. On the other hand, other TKIs such as vatalanib and gefitinib (respective VEGFR and EGFR inhibitors) did not induce hypertension in treated mice [15].

Our results did not show differences in heart rate between control and rats treated with Su for 21 days. Similar results were found by Blasi et al. [29] in rats in the first 28 days of treatment with Su. On the other hand, other studies reported either increased [14] or decreased [32,33] heart rate following Su treatment, which suggests that these differences might be related to changes in dosing and/or duration of treatment.

The hypertensive mechanisms related to Su therapy might be directly associated with its inhibitor effect on vascular endothelial growth factor (VEGF) signal pathways, leading to density-reduced microvessels, reduced NO production associated with endothelial dysfunction, and enhancement of oxidative stress [14]. A reduction in NO bioavailability might also be due to an imbalance between vasodilator (NO) and vasoconstrictor (ET-1) molecules in favor of the latter [9]. In addition, previous studies in humans reported that polymorphisms of genes such as VEGFA, VEGFR-2, ET-1, and eNOS might predispose to hypertension after Su treatment [34]. Our studies demonstrated a reduction in endothelium-dependent vasodilation in treated animals when compared with normotensive control animals, an observation that was abolished after the addition of NOX1 inhibitor ML171. This finding suggests a major contribution of NOX1 isoform in the endothelial dysfunction induced by Su. The presence of L-NAME (an inhibitor of eNOS) produced a lower response to ACh in both experimental groups up to a similar extent, and no changes were observed with endothelium-independent vasorelaxation with SNP. Similar results were observed by Thijs et al. [27] and Neves et al. [15] in mesenteric arteries from Su-treated rats and mesenteric arteries from mice incubated with vatalanib, respectively. Nevertheless, the incubation of mice mesenteric arteries with EGFR inhibitor gefitinib did not show changes in the vascular response to ACh [15].

In agreement with our results, Thijs et al. [25] found that the differences observed in the ACh-induced response between control and Su-treated animals was eliminated in the presence of L-NAME. This indicates that a reduction in NO bioavailability in Su-treated rats might be responsible for the high blood pressure observed in these animals. However, since the presence of L-NAME produced a further slight decrease in ACh-induced vasorelaxation than that observed with Su treatment alone, factors other than NO might also play a role in the genesis of hypertension in the context of Su treatment; these factors include oxidative stress, ET-1, and gene polymorphisms [15,34]. The fact that endothelium-independent vasorelaxation was unaffected indicates that Su effect on vascular function occurs in an endothelium-dependent manner.

Structural vascular modifications were also found in our study. Thus, thickness, CSA, and media/lumen ratio were significantly increased in thoracic aortas from Su-treated rats. Regarding the classification described by Renna et al. [35], these results indicate that severe hypertrophic vascular remodeling appears in the aorta of Su-treated rats when compared with control animals. These results are in agreement with previous studies in mesenteric arteries from mice treated with vatalanib, in which an increased media/lumen ratio was exhibited [15]. On the other hand, the use of gefitinib had no effect on cross-sectional area [15]. Reductions in the diameter and vasoconstriction of mice [27] and rat [11] mesenteric arteries, together with stiffness and fibrosis in human large arteries [12], have been observed after treatment with Su and other VEGF inhibitors.

Vascular wall remodeling processes involve changes in growth and migration of VSMCs, endothelial dysfunction, inflammatory processes, and changes in extracellular matrix components (EMCs) [35]. In this way, collagen types I and III are the main isoforms in adventitia [36], while collagen I, III, and V are mainly present in media layer [37]. Positive staining for Col1α1 immunofluorescence was found in the adventitia and media layer of both groups of animals. In the media layer from the Su-treated animals, the staining of Col1α1 was intense compared with control rats. However, only in the intima layer of Su-treated rats, intense Col1α1 staining was found. This difference of Col1α1 staining between both groups of animals might be due to changes in the collagen composition as a consequence of the treatment of Su since modifications in the quantity and location of collagen fibers seem to appear in aorta diseases [38]. On the other hand, there is no evidence concerning the presence of collagen type I in the inner layer. However, the deposition of collagen type I was observed in our study in the basement membrane of Su-treated aorta when compared with the control group. These collagen fibers might interpose between endothelial cells and the middle layer, thus causing an increase in aorta thickness. The increase in Col1α1 staining observed in rats subjected to Su treatment is supported by the enhancement in the total content of collagen and upregulation of profibrotic cytokine TGF-β1 and collagen type I in these animals. Previous studies in our lab showed a rise in the expression of collagen type I, TGF-β1, and tissue inhibitor matrix metalloproteinases (TIMP-1) accompanied by a decrease in that of matrix metalloproteinase (MMP-9) in heart tissue from Su-treated rats [14]. Based on these results, changes in enzymes involved in the turnover of collagen might also be affected in aortas from rats treated with Su.

As previously reported, oxidative stress is one of the mechanisms involved in the synthesis of ECM and accumulation of collagen fiber in the intima layer during the process of vascular remodeling [39,40], where an increase of pro-oxidant species together with a reduction in endothelial NO production is observed [41]. The most widely used method to quantify cellular O_2_^−^ in mammalian cells is based on DHE staining. MitoSOX or HPLC are currently being used for mitochondrial O_2_^−^ detection, although one major limitation of the latter is the inability to distinguish the specific cells contributing to ROS production in response to pathological stimuli [42]. In our study, we found an increase in superoxide anion (O_2_^−^) content in the aorta of rats treated with Su compared with the control group. Interestingly, the use of commercially available inhibitors of NOX isoforms, namely ML171, GKT136901, and VAS2870, resulted in clearly visible and quantifiable reductions of O_2_^−^ production in aortas from Su-treated rats. Moreover, when NADPH oxidase activity (a pivotal enzyme for ROS production in the hypertensive context) was determined by means of lucigenin-enhanced chemiluminescence, an increase in the activity was found in aortas from Su-treated rats when compared to the control group, which was also blocked via specific NOX inhibition. In contrast, the eNOS inhibitor, L-NAME, did not affect superoxide release in Su-treated group. Taken together, these results revealed NOX1 as the main isoform involved in the production of ROS in aorta from Su-treated animals, thus confirming the results concerning endothelium-dependent vasodilation.

Neves et al. [15] previously demonstrated that vatalanib treatment induced activation of NADPH oxidase in human aortic endothelial cells (HAECs) and mice kidney. However, the reported changes were found following treatment with gefitinib, where the increased NADPH-dependent O_2_^−^ generation in HAECs by vatalanib is inhibited by a NOX1/4 inhibitor [15].

In additional experiments, we also found an enhancement in the protein expression of 3-nitrotyrosine, a stable byproduct whose production is modified in the context of oxidative stress [42], in the aorta from Su-treated rats compared to the control group. Regarding specific changes in the expression of NOX isoforms of NADPH oxidase, NOX1, NOX2, and NOX4 were upregulated in Su-treated rats, although NOX4 showed no significant changes in the protein expression when compared with non-treated animals. Previous studies also found modifications in the regulation of NOX isoforms after treatment with such TKIs. An increase in mRNA expression of NOX1, together with a decrease in the expression of NOX4, was found in heart and kidney, respectively, from mice treated with vatalanib and gefitinib when compared with control animals, accompanied by no alterations in NOX2 levels in both tissues. When studies were carried out in vascular smooth muscle cells and HAEC, differences were observed depending on the NOX isoforms and the source of TKI added in the incubation medium [15]. In addition, an increase in NOX expressions was observed in the heart of rats treated with Su [14]. All these findings suggest differential effects of TKIs on NADPH system; however, despite specific differences observed in NOX isoform expression, an oxidative imbalance is highly involved in arterial hypertension and toxicity produced by Su and other TKIs, including VEGFR and EFGR inhibitors.

In our study, the presence of redox imbalance was supported by decreased NO levels measured in aortas from Su-treated animals, where the use of NOX inhibitors increased the bioavailability of NO over those values measured in the control group. These results concerning NO concentration parallel the pattern observed for eNOS protein expression and activation, although animals subjected to treatment with Su also showed an upregulation of total eNOS gene expression, interestingly.

## 5. Conclusions

Our study provides novel insights on the molecular mechanisms involved in the vascular damage produced by treatment with the tyrosine kinase inhibitor, sunitinib (Su). As expected, Su administered daily for three weeks led to development of arterial hypertension, which might be due to endothelial dysfunction and vascular remodeling induced by the drug via overactivation of the NADPH oxidase system (mainly via NOX 1 isoform) and, consequently, an alteration of the redox balance. We must note, however, that the current data cannot ascertain whether arterial hypertension is secondary to oxidative stress or due to direct vascular injury following Su treatment. In this sense, previous studies in humans treated with Su demonstrated that endothelial dysfunction might be relevant in maintaining (or further increasing) high arterial pressure but does not precede its initial development [27]. Another limitation of our study is that histological and biochemical disturbances have been reported in aortic tissue from Su-treated rats; further studies on resistant vessels (e.g., mesenteric arteries), will probably help elucidate those vascular molecular mechanisms involved in Su-associated arterial hypertension.

## Figures and Tables

**Table 1:** The primers used for real-time RT-PCR.

Gene	Forward (5′–3′)	Reverse (5′–3′)
Col1	TCAGGGGCGAAGGCAACAGT	TTGGGATGGAGGGAGTTTACACGA
TGF-β1	GCCCTGGATACCAACTACTGCT	AGGCTCCAAATGTAGGGGCAGG
NOX1	TTCACCAATTCCCAGGATTGAAGTGGATGGTC	GACCTGTCACGATGTCAGTGGCCTTGTCAA
NOX2	CCCTTTGGTACAGCCAGTGAAGAT	CAATCCCAGCTCCCACTAACATCA
NOX4	TTGCTTTTGTATCTTC	CTTACCTTCGTCACAG
eNOS	GGGCCAGGGTGATGAGCTCTG	CCCTCCTGGCTTCCAGTGTCC
GAPDH	GCCAAAAGGGTCATCATCTCCGC	GGATGACCTTGCCCACAGCCTTG

Notes: Collagen type I (Col1), transforming growth factor β (TGF-β1), NADPH oxidase isoforms (NOX1, NOX2, and NOX4), endothelial nitric oxide synthase (eNOS), and glyceraldehyde 3-phosphate dehydrogenase (GAPDH).
